# We’ll take it from here: Veterans Affairs OPAT management of home IV therapy initiated at community hospitals

**DOI:** 10.1017/ash.2023.476

**Published:** 2023-07-06

**Authors:** Allison R. Tiemann, Jessica M. Guastadisegni, Marcus A. Kouma, Kevin C. Kelly, Reuben J. Arasaratnam, Donald F. Storey

**Affiliations:** 1 Veterans Affairs North Texas Health Care System, Dallas, TX, USA; 2 Veterans Affairs North Texas Health Care System and University of Texas Southwestern Medical Center, Dallas, TX, USA

## Abstract

An outpatient parenteral antimicrobial therapy team from a Veterans Affairs facility managed patients discharged from their own facility and neighboring community hospitals. There were no significant differences in adverse outcomes between the groups, but a majority of regimens were modified from those initially proposed by community providers.

## Introduction

Patients have benefited for decades from the convenience and lower costs afforded by outpatient parenteral antimicrobial therapy (OPAT) without sacrificing quality.^1,2^ Within the Veterans Health Administration (VHA), OPAT orders are typically approved by a member of the facility antimicrobial stewardship team.^3^ This oversight includes veterans discharged from VHA and community, non-VHA facilities. For community facility discharges, VHA staff are also called upon to interface with community providers, navigating the veteran through a complex and sometimes fraught transition of care process. While other VHA facilities have reported outcomes and identified stewardship opportunities from their OPAT programs,^4–9^ none have compared VHA facility-managed OPAT discharges from the community to those from their own facility.

At the Veterans Affairs North Texas Health Care System (VANTHCS), the OPAT program is led by members of the facility’s antimicrobial stewardship team (three ID pharmacists, one ID physician, and one rotating ID fellow) and reports to the antimicrobial stewardship subcommittee. The OPAT team reviews all community hospital OPAT requests and either assumes responsibility for follow-up of the veteran’s therapy (sometimes with modifications), authorizes the veteran to continue with the community infectious diseases provider, or recommends transfer to VANTHCS for further evaluation (Supplementary Figure 1). The determination of OPAT candidacy for patients discharging from VANTHCS is primarily made by the ID fellow and attending rounding on the ID consult service, both of whom have knowledge of the OPAT program. Once established on the OPAT service, a pharmacist calls veterans weekly to screen for potential adverse drug events and reviews laboratory results. In addition, a VANTHCS ID clinic appointment is requested as feasible for veterans prior to the end of therapy date. The goal of this study was to compare the incidence of unplanned hospitalization, emergency department visits, reinitiation of antimicrobials, and adverse events for OPAT discharges from VANTHCS versus community hospitals. Additionally, we sought to characterize changes in antimicrobial therapy made by the VANTHCS OPAT team at the time veterans entered the OPAT program from community hospitals.

## Methods

We performed a retrospective chart review with the intention of including all 367 adult veterans enrolled in the VANTHCS OPAT program between October 1, 2020, and September 30, 2022. Due to limited time and project resources, for enrollments after July 9, 2021, only community hospital discharges were included so that both groups were comprised of at least 100 veterans. Patients were excluded if they did not receive at least one dose of intravenous (IV) antimicrobials on OPAT or follow-up by the OPAT team. Veterans needing IV antimicrobials who were transferred to VANTHCS from a community hospital or transferred to a community hospital from VANTHCS were excluded. Patients followed by community hospital providers after discharge were also excluded.

Clinical data were collected via manual review of patient charts in the Computerized Patient Record System used at VANTHCS. Data retrieved included patient demographics, comorbidities, indications for OPAT, antimicrobial recommendations and orders, planned and actual treatment durations, pertinent laboratory values, and documentation of other patient-specific factors related to infection. All documentation related to OPAT initiation, monitoring, and follow-up were reviewed to determine the outcome of treatment.

The primary outcome was a composite of unplanned hospital readmissions, emergency department visits, reinitiation of antimicrobials (new order for antimicrobial(s) for the same indication) within 30 days of actual end of treatment, and death from any cause while on OPAT. Secondary outcomes included the individual components of the primary outcome, adverse drug events (any event resulting in discontinuation or dose change of one or more agent(s) prescribed for OPAT; abnormal laboratory values; and patient self-reported events determined by the OPAT team as potentially medication-related), and IV access complications. Changes made by the VANTHCS OPAT team were categorized as follows: spectrum, defined as a difference in the point value of one antibiotic regimen compared to another based on the Antibiotic Spectrum Index^10^; duration, defined as any increase or decrease in the planned length of therapy; route of administration, defined as a switch from IV to oral or oral to IV; convenience, defined as a decrease in the number of infusions per day and/or use of an alternative agent to avoid the need for therapeutic drug monitoring.

Descriptive statistics were used for the demographic and baseline clinical data. All analyses for categorical data were performed using Fisher’s exact test; continuous variables were compared using the Student’s *t* test or Mann–Whitney U test. Statistical significance was set at *p* <0.05. The VANTHCS Institutional Review Board approved the study. Based on the study design, informed consent was not required.

## Results

A total of 262 out of 376 OPAT enrollments during the 2-year period were reviewed; of these, 61 were excluded, and 201 were included in the final data analysis. Of those included, 101 were discharged to OPAT from VANTHCS and 100 from community hospitals (Supplementary Figure 2). Aside from a higher mean Charlson comorbidity index score in the VHA group (5.8; SD, 3.3 vs. 4.5; SD, 2.6; *P* = .004) and a greater number of prosthetic joint infections in the community hospital group (0/101, 0.0% vs. 23/100, 23.0%; *P* < .001), the groups were similar at baseline (Table 1). Of note, the frequencies of methicillin-sensitive (22/101, 21.8% vs. 22/100, 22.0%; *P* = 1) and -resistant (9/101, 8.9% vs. 9/100, 9.0%; *P* = 1) *Staphylococcus aureus* infections between the two groups were identical. The primary composite outcome occurred in 68/201 (33.8%) patients overall, and there were no significant differences between the VHA (37/101, 36.6%) and community hospital (31/100, 31.0%) groups (Table 2).

**Table 1.** Baseline veteran characteristics for VHA and community hospital OPAT discharges

Characteristic^a^	VHA (*N* = 101)	Community (*N* = 100)	*P*-value
Age, mean y (±SD)	65.8 (±11.2)	64.0 (±10.7)	.09
Male sex	94 (93.1)	98 (98.0)	.17
Antibiotic allergies ≥1	17 (16.8)	22 (22.0)	.38
Charlson comorbidity index, mean (± SD)	5.83 (±3.3)	4.46 (±2.6)	.004
**Race**			
White	69 (68.3)	69 (69.0)	1
African American	26 (25.7)	20 (20.0)	.40
Other	2 (2.0)	2 (2.0)	1
Unknown	4 (4.0)	9 (9.0)	.16
**County of residence**			
Dallas	36 (35.6)	16 (16.0)	.002
Surrounding^b^	42 (41.6)	62 (62.0)	.005
Other	23 (22.8)	21 (21.0)	.86
**Site of infection** ^c^			
Bacteremia	35 (34.7)	25 (25.0)	.17
Endocarditis	7 (6.9)	1 (1.0)	.06
Genitourinary	5 (5.0)	12 (12.0)	.08
Intra-abdominal	3 (3.0)	8 (8.0)	.13
Osteomyelitis	42 (41.6)	31 (31.0)	.14
Pneumonia	4 (4.0)	1 (1.0)	.37
Prosthetic joint	0 (0)	23 (23.0)	<.001
Septic arthritis	5 (5.0)	5 (5.0)	1
Skin and soft tissue	8 (7.9)	8 (8.0)	1
Other	21 (20.8)	14 (14.0)	.26
**Causative organism** ^c^			
Enterobacterales order	21 (20.8)	28 (28.0)	.25
*Enterococcus* species	12 (11.9)	4 (4.0)	.07
Methicillin-resistant *Staphylococcus aureus*	9 (8.9)	9 (9.0)	1
Methicillin-sensitive *Staphylococcus aureus*	22 (21.8)	22 (22.0)	1
*Pseudomonas* species	8 (7.9)	4 (4.0)	.37
*Streptococcus* species	19 (18.8)	13 (13.0)	.34
Other	48 (47.5)	45 (45.0)	.78
Not available^d^	15 (14.9)	19 (19.0)	.46
**Rationale for therapy selection**			
Targeted	83 (82.2)	77 (77.0)	.39
Empiric	18 (17.8)	23 (23.0)	.39

Note. VHA, Veterans Health Administration; OPAT, outpatient parenteral antimicrobial therapy; SD, standard deviation.

a All data represented as no. (%) unless otherwise noted.

b Surrounding counties include Collin, Denton, Ellis, Kaufman, Rockwall, and Tarrant.

c Patients may have one or more site(s) of infection or causative organism(s).

d Culture-negative or not available.

**Table 2.** Veteran outcomes following VHA and community hospital OPAT discharges

Outcome	VHA (*N* = 101), No. (%)	Community (*N* = 100), No. (%)	*P*-value
**Composite** ^a^	37 (36.6)	31 (31.0)	.46
**Individual**			
Unplanned readmission	25 (24.8)	18 (18.0)	.30
ED visit	33 (32.7)	27 (27.0)	.44
Reinitiation of antimicrobials	12 (11.9)	12 (12.0)	1
Death	3 (3.0)	0 (0.0)	.25
**IV access complications**			
Loss of access	6 (5.9)	5 (5.0)	1
DVT	1 (1.0)	0 (0.0)	1
Infection	0 (0.0)	0 (0.0)	…
Other	6 (5.9)	3 (3.0)	.50
**Adverse drug events**			
Diarrhea	7 (6.9)	7 (7.0)	1
Nausea/vomiting	6 (5.9)	3 (3.0)	.50
CPK > upper limit of normal^b^	4 (4.0)	3 (3.0)	1
Elevated LFT(s)^c^	0 (0.0)	3 (3.0)	.12
AKI^d^	2 (2.0)	1 (1.0)	1
Rash/pruritus	1 (1.0)	3 (3.0)	.37
CNS effects	2 (2.0)	3 (3.0)	.68
Other	4 (4.0)	7 (7.0)	.37

Note. VHA, Veterans Health Administration; OPAT, outpatient parenteral antimicrobial therapy; SD, standard deviation; ED, emergency department; IV, intravenous; DVT, deep venous thrombosis; CPK, creatine phosphokinase; LFTs, liver function tests; AKI, acute kidney injury; CNS, central nervous system.

a Composite of unplanned readmission, ED visit, reinitiation of antimicrobials, and death.

b Upper limit of normal determined by reference range of the reporting laboratory.

c Aspartate transaminase, alanine transaminase, alkaline phosphatase, and bilirubin included.

d Increase in serum creatinine by ≥0.3 mg/dL within 48 h or 1.5 times baseline, which is known or presumed to have occurred in the prior 7 d.

## Discussion

According to a recent national VHA survey of antimicrobial stewardship practices, 50% of facility respondents reported working with community providers to supply medications without a face-to-face visit, and only 25% reported scheduling a clinic appointment for veterans prior to approving OPAT requests from community providers.^3^ By demonstrating similar outcomes for veterans discharging from VHA and community facilities, the present study is the first to lend support to the widely adopted practice of approving OPAT orders from information gleaned exclusively from medical record review and telephone calls with community providers and without a prior in-person assessment. Our results are similar to another VHA OPAT study reported by Guenther et al,^9^ and to a previous study conducted at our facility from 2009 to 2012 when the reported rate of unplanned hospital readmission within 30 days was found to be 21% overall,^7^ identical to the present study.

Although there were no significant differences in outcomes identified between the two groups, the VHA group had a higher burden of comorbidities (as indicated by the significantly higher Charlson comorbidity index in that group). We believe this difference is reflective of the judicious and risk-averse selection process we followed to ensure safe transitions of care from the community hospital into our OPAT program. Specifically, veterans with more, or more significant, comorbidities may have been deemed poor candidates for direct discharge from the community hospital into our program without an in-person evaluation by our ID team. While this may introduce selection bias in favor of the community hospital group (in that they are, overall, less sick), it provides important context for the interpretation of our findings—namely that a detailed assessment of comorbidities is a mandatory part of determining OPAT candidacy. As a further testament to our review process, safety- and stewardship-conscious treatment modifications were recommended in more than half of community hospital cases (Supplementary Table 1), though the ultimate impact of those changes on outcomes was not evaluated in this study.

Limitations include the retrospective, single-center design, and the potentially underpowered nature of this study from a small sample size. We used the Charlson comorbidity index to attempt to compare the overall health of the two groups, but its generalizability to shorter timeframes and specific populations, as in the present study, is uncertain. The observed rates for the primary and secondary outcomes are affected by the unavailability of documentation from outside hospitals, which is likely to affect the community hospital group more and is a potential source of bias. Lastly, additional selection bias may have been introduced because not all enrolled study patients were included in the analysis. Despite these limitations, our findings may inform future VHA stewardship surveys, policy directives, and/or OPAT research regarding the management of requests from community providers.

## Supporting information

Tiemann et al. supplementary material Tiemann et al. supplementary material

## Supplementary material

For supplementary material accompanying this paper visit http://doi.org/10.1017/ash.2023.476. click here to view supplementary material
