# Supplementary material for: We’ll take it from here: Veterans Affairs OPAT management of home IV therapy initiated at community hospitals
Source: Antimicrob Steward Healthc Epidemiol. 2023 Jul 6;3(1):e197. doi: 10.1017/ash.2023.476 (PMC10654944; doi:10.1017/ash.2023.476)
Supplement: Tiemann et al. supplementary material [file S2732494X2300476Xsup001.docx]

**Supplementary Figure 1**

VA North Texas Health Care System (VANTHCS) process for reviewing community hospital requests for outpatient parenteral antimicrobial therapy (OPAT)


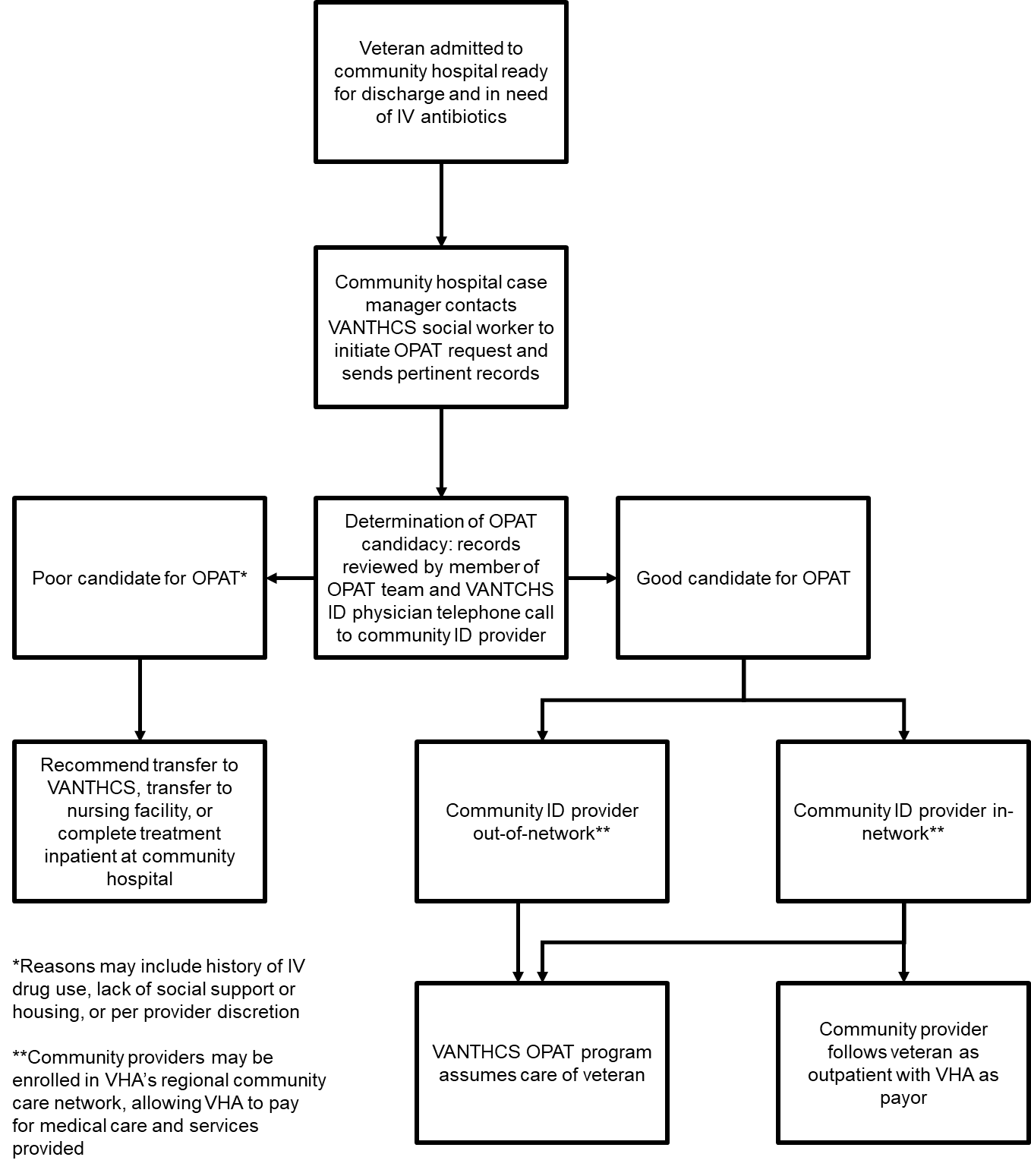


**Supplementary Figure 2**

Overview of patient charts reviewed for inclusion/exclusion


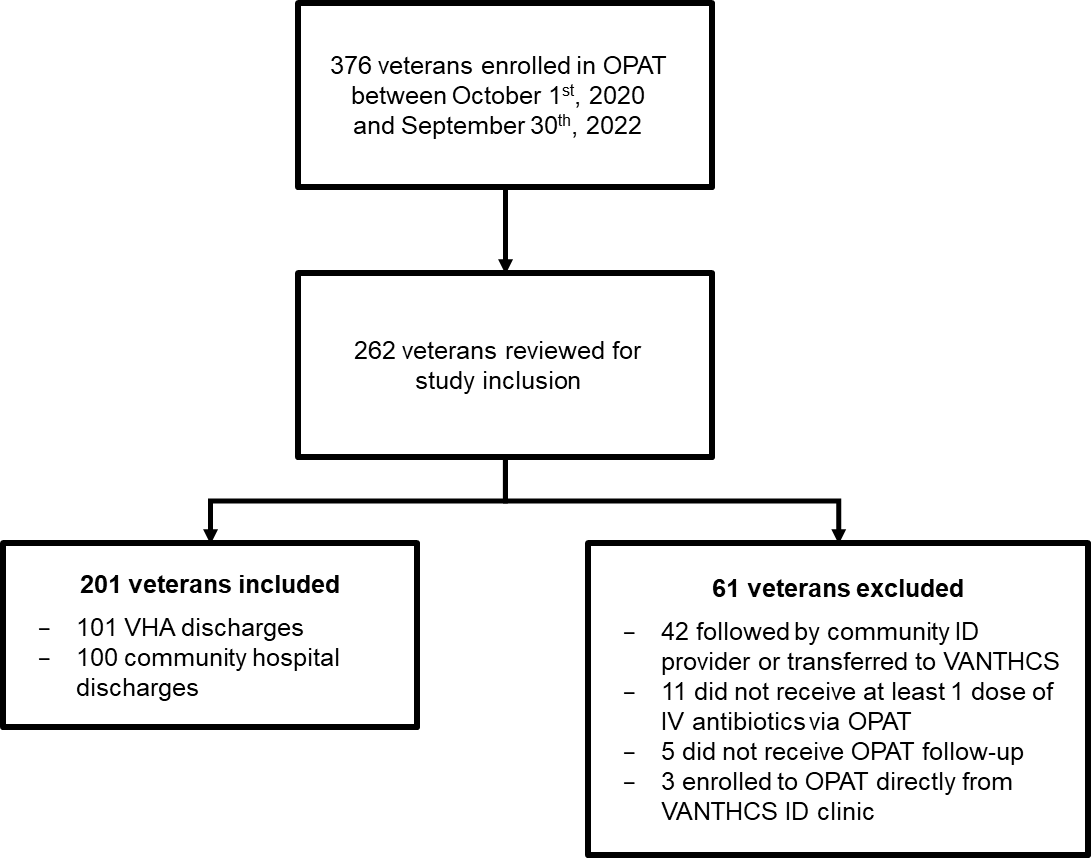


**Supplementary Table 1**

| **VANTHCS OPAT Program Changes Made to Antibiotic Regimens Planned by Community Hospitals** | |
| --- | --- |
| No change | 39 |
| Spectrum | 36 |
| Escalation | 15 |
| De-escalation | 21 |
| IV-to-PO | 3 |
| Duration | 13 |
| Convenience | 48 |
| Note: 100 total changes recommended across 61 patients | |
